# Skin toxicity and effectiveness of first-line enfortumab vedotin plus pembrolizumab for urothelial carcinoma: a prospective multicenter real-world study in Japan

**DOI:** 10.1016/j.esmorw.2026.100804

**Published:** 2026-10-17

**Authors:** K. Tamura, T. Tsujino, R. Taoka, K. Kobayashi, M. Miyake, K. Ogawa, Y. Sasaki, S. Tatarano, S. Katayama, M. Kato, H. Fukuhara, Y. Abe, Y. Oda, R. Minato, R. Shigehisa, R. Maenosono, M. Sugimoto, K. Shiraishi, K. Fujimoto, K. Wada, J. Furukawa, H. Enokida, M. Araki, J. Uchida, K. Inoue, T. Inamoto, West Japan Uro-oncology Collaboration Group

**Affiliations:** 1 Department of Urology, Hamamatsu University School of Medicine, Hamamatsu, Japan; 2 Department of Urology, Faculty of Medicine, Osaka Medical and Pharmaceutical University, Takatsuki, Japan; 3 Department of Urology, Faculty of Medicine, Kagawa University, Kagawa, Japan; 4 Department of Urology, Graduate School of Medicine, Yamaguchi University, Ube, Japan; 5 Department of Urology, Nara Medical University, Kashihara, Japan; 6 Department of Urology, Shimane University Faculty of Medicine, Izumo, Japan; 7 Department of Urology, Tokushima University Graduate School of Biomedical Sciences, Tokushima, Japan; 8 Department of Urology, Graduate School of Medical and Dental Sciences, Kagoshima University, Kagoshima, Japan; 9 Department of Urology, Graduate School of Medicine, Dentistry and Pharmaceutical Sciences, Okayama University, Okayama, Japan; 10 Department of Urology, Osaka Metropolitan University Graduate School of Medicine, Osaka, Japan; 11 Department of Urology, Kochi Medical School, Kochi University, Kochi, Japan

**Keywords:** enfortumab vedotin, immortal time bias, pembrolizumab, progression-free survival, skin toxicity, urothelial carcinoma

## Abstract

**Background:**

Skin toxicity is a common adverse event associated with enfortumab vedotin plus pembrolizumab (EVP) but its association with treatment outcomes remains unclear. We aimed to evaluate this association in patients with locally advanced or metastatic urothelial carcinoma (la/mUC).

**Patients and methods:**

This prospective multicenter observational study conducted in Japan enrolled patients receiving first-line EVP. Skin toxicity incidence, onset, and management were evaluated. Objective response rates (ORRs) were compared according to the development of skin toxicity within 42 days. Progression-free survival (PFS) was assessed using a prespecified 42-day landmark analysis and a time-dependent Cox model to account for immortal time bias.

**Results:**

Among 163 assessable patients, 94 (57.7%) developed skin toxicity at a median of 13 days, of whom 81 (86.2%) recovered. Patients with skin toxicity within 42 days had a higher ORR than those without (70.1% versus 44.2%, *P* < 0.001). In the landmark cohort (*n* = 144), skin toxicity was associated with longer PFS [hazard ratio (HR) 0.43, 95% confidence interval (CI) 0.22-0.84, log-rank *P* = 0.011]. In multivariable time-dependent Cox analysis, skin toxicity remained independently associated with longer PFS (HR 0.44, 95% CI 0.23-0.83, *P* = 0.011).

**Conclusion:**

Skin toxicity associated with first-line EVP was frequent, occurred early, and was generally manageable. Its development was associated with a higher ORR and its association with longer PFS persisted after accounting for immortal time bias. Skin toxicity may serve as a potential on-treatment prognostic indicator of favorable outcomes, although further validation is warranted.

## Highlights

• Skin toxicity occurred in 57.7% of patients with UC, with a median onset of 13 days.
• Most skin toxicities were manageable but severe events were also observed.
• Patients with skin toxicity within 42 days had a higher ORR.
• Skin toxicity was associated with longer PFS after accounting for immortal time bias.
• Skin toxicity may provide on-treatment prognostic indicator during first-line EVP.

## Introduction

The therapeutic landscape for locally advanced or metastatic urothelial carcinoma (la/mUC) has undergone a paradigm shift with the advent of enfortumab vedotin (EV) plus pembrolizumab (EVP) combination therapy.^1^ The pivotal phase III EV-302/KEYNOTE-A39 trial demonstrated that first-line EVP nearly doubled the median overall survival (OS) compared with traditional platinum-based chemotherapy [33.8 versus 15.9 months; hazard ratio (HR) 0.51].^2^ Furthermore, median progression-free survival (PFS) was longer with EVP (12.5 versus 6.3 months; HR 0.48). Consequently, the EVP combination has been rapidly established as the new standard of care for first-line treatment of la/mUC.

Despite its remarkable clinical benefits, EVP therapy is frequently associated with unique adverse events that require careful management.^3^ Skin toxicity is the most common adverse event. In the EV-302 trial, the incidence of skin toxicity associated with EVP was 66.8% for any grade, with 15.5% of patients experiencing grade ≥3 reactions.^1^ Consistent with these findings, recent real-world cohorts reported any-grade skin toxicity in 57.0%-60.4% of patients receiving EVP, with severe events observed in up to 18.8% of cases.^4^^,^^5^ These skin reactions typically emerge early, often within the first 3 months of treatment. Notably, the median time to onset has been reported to be as early as 0.5 months in patients receiving EV monotherapy.^6^ Although most cases are manageable with appropriate dermatological interventions, severe toxicity may necessitate dose modification or permanent discontinuation, representing a substantial clinical challenge.^1^^,^^4^^,^^5^

Emerging evidence suggests that skin toxicity is associated with treatment efficacy. Several retrospective cohort studies have consistently demonstrated that skin toxicity is associated with improved clinical outcomes, including PFS, OS, and objective response rates (ORRs).^6^, ^7^, ^8^ These findings suggest that skin toxicity may reflect drug exposure and on-target activity in nectin-4-expressing tissues.^9^

However, whether this toxicity–outcome association holds true in the setting of EVP combination therapy remains unclear. Although a few retrospective cohort studies have explored this relationship, their findings have been heterogeneous; initially positive associations often did not persist after rigorous statistical adjustment for confounding factors, such as treatment duration.^4^ Robust evidence demonstrating the effect of skin toxicity on the clinical outcomes of EVP therapy is currently lacking. Therefore, we conducted this prospective observational study to evaluate the association of skin toxicity with PFS and ORR in Japanese patients receiving first-line EVP, thereby clarifying its potential role as an on-treatment prognostic indicator in a real-world setting. The association between skin toxicity and OS was also evaluated as an exploratory endpoint.

## Patients and methods

### Study design and patients

This prospective, multicenter study was conducted across 11 institutions of the West Japan Uro-oncology Collaboration Group. We enrolled patients with la/mUC who initiated first-line EVP between 1 October 2024 and 31 March 2026. All participants provided written informed consent. The analysis included data available through 15 May 2026. The study protocol was centrally reviewed and approved by the Ethics Committee of Kagawa University (approval no. 2024-161) and institutional authorization was obtained at each participating center. The study was conducted in accordance with the Declaration of Helsinki and all data used for analysis were deidentified.

### Treatment protocol

Patients received EV (1.25 mg/kg i.v. on days 1 and 8) plus pembrolizumab (200 mg i.v. on day 1) every 3 weeks according to the standard regimen.^1^ The initial EV dose could be reduced at the physician’s discretion based on patient characteristics (e.g. age, performance status, and comorbidities). Treatment was continued until disease progression, initiation of subsequent therapy, unacceptable toxicity, or withdrawal of consent. Dose modifications were permitted for adverse events according to institutional practice and the pivotal trial safety recommendations.

### Clinical evaluation

Baseline clinical data, including demographic characteristics, a medical history, Eastern Cooperative Oncology Group performance status (ECOG PS), and laboratory evaluations of organ function, were recorded before EVP initiation. Baseline disease staging was carried out within 28 days before EVP initiation using contrast-enhanced computed tomography (CT), or noncontrast CT in patients with severe renal impairment, together with bone scintigraphy. Target lesions were documented according to the RECIST, (version 1.1, RECIST Working Group, EORTC Data Centre, Brussels, Belgium).^10^ Tumor responses were assessed without an independent central radiologic review. The initial follow-up imaging was scheduled on day 42, immediately before the third treatment cycle. Subsequent imaging assessments during treatment were carried out at the discretion of the treating physicians as part of routine clinical practice, typically every 8-12 weeks.

Skin toxicity included cutaneous adverse events considered clinically related to EVP treatment and was graded according to the National Cancer Institute Common Terminology Criteria for Adverse Events (CTCAE), (version 5.0, National Cancer Institute (NCI), Bethesda, MD). Skin toxicity was assessed and graded primarily by the treating physicians at each participating institution. Dermatological consultation was obtained for selected cases, particularly for severe skin toxicity or when systemic corticosteroid treatment was considered. Individual clinical phenotypes of skin toxicity and attribution to EV or pembrolizumab were not systematically collected. Recovery from skin toxicity was defined according to the maximum event severity: for grade ≥2 events, recovery was considered improvement to grade ≤1; for grade 1 events, recovery included complete resolution (grade 0) or partial resolution with clinical improvement. This definition was used because, given the mild nature of grade 1 toxicity, partial improvement was considered clinically meaningful even without complete resolution.

### Study endpoints

The primary endpoint was PFS, defined as the time from initiation of EVP therapy to documented disease progression or death from any cause. Patients without a PFS event were censored at the initiation of subsequent anticancer treatment, consolidative surgery, or last clinical follow-up. Patients undergoing consolidative surgery were censored at the date of surgery because surgery represented a change in treatment strategy in the absence of documented disease progression.

Secondary endpoints included ORR and safety, with a particular focus on skin toxicity, including its incidence, severity, time to onset, recovery rate, and time to recovery. ORR was defined as the proportion of patients who achieved a complete response (CR) or partial response (PR) as the best overall response according to RECIST, version 1.1. Safety endpoints were evaluated based on adverse events graded according to CTCAE, version 5.0.

OS, defined as the time from initiation of EVP therapy to death from any cause, was evaluated as an exploratory endpoint. Patients alive at the last clinical follow-up were censored at that date.

### Statistical analysis

Patients were classified according to skin toxicity development within the first 42 days of EVP therapy. Baseline characteristics and ORR were compared using the Mann–Whitney *U* test for continuous variables and the two-sided Fisher’s exact test for categorical variables.

A prespecified 42-day landmark analysis was carried out to minimize immortal time bias.^11^ This time point corresponded to initial radiographic evaluation and encompassed most skin toxicity events. Patients with a PFS event or censoring before day 42 were excluded from the PFS landmark analysis, whereas those who died or were censored before day 42 were excluded from the OS landmark analysis. PFS and exploratory OS were estimated using the Kaplan–Meier method and compared using the log-rank test. As a sensitivity analysis, ORR was compared among patients included in the 42-day PFS landmark cohort. An exploratory analysis of PFS according to skin toxicity severity (none, grade 1-2, or grade ≥3) was also carried out in this cohort. HRs and 95% confidence intervals (CIs) for skin toxicity were estimated using univariable Cox proportional hazards models.

Additionally, a time-dependent Cox proportional hazards model was used, with the occurrence of skin toxicity treated as a time-varying covariate, as this approach is considered optimal for minimizing immortal time bias in studies evaluating treatment-emergent events.^12^ HRs and 95% CIs were calculated. The multivariable Cox model for PFS included skin toxicity and the following four clinically relevant covariates selected a priori: ECOG PS, liver metastasis, prior adjuvant nivolumab, and histology (pure UC versus variant histology). As a sensitivity analysis, the initial EV dose was additionally included in the multivariable model. Given the limited number of deaths, the association between skin toxicity and OS was evaluated using a univariable time-dependent Cox regression model without multivariable adjustment. Analyses were carried out using EZR software (Saitama Medical Center, Jichi Medical University, Saitama, Japan).^13^ Statistical significance was defined as a two-sided *P* < 0.05. The reporting of this study was guided by the ESMO Guidance for Reporting Oncology real-World evidence.^14^

## Results

### Patient disposition and baseline characteristics

Patient disposition is shown in Figure 1. Of the 172 patients who initiated EVP, 9 receiving subsequent-line therapy were excluded, leaving 163 patients in the overall cohort. This cohort was used to evaluate the clinical profile of skin toxicity and carry out time-dependent Cox regression analysis. For the analysis of baseline characteristics and ORR, patients were classified according to the development of skin toxicity within 42 days: 77 with skin toxicity and 86 without skin toxicity. Nineteen patients with a PFS event or censoring before day 42 were excluded from the PFS landmark analysis (PFS landmark cohort, *n* = 144). The median follow-up was 7.1 months [interquartile range (IQR), 4.0-10.5 months]. During follow-up, nine patients underwent consolidative surgery (radical cystectomy or nephroureterectomy) after achieving a response or stable disease with EVP.

**Figure 1:**
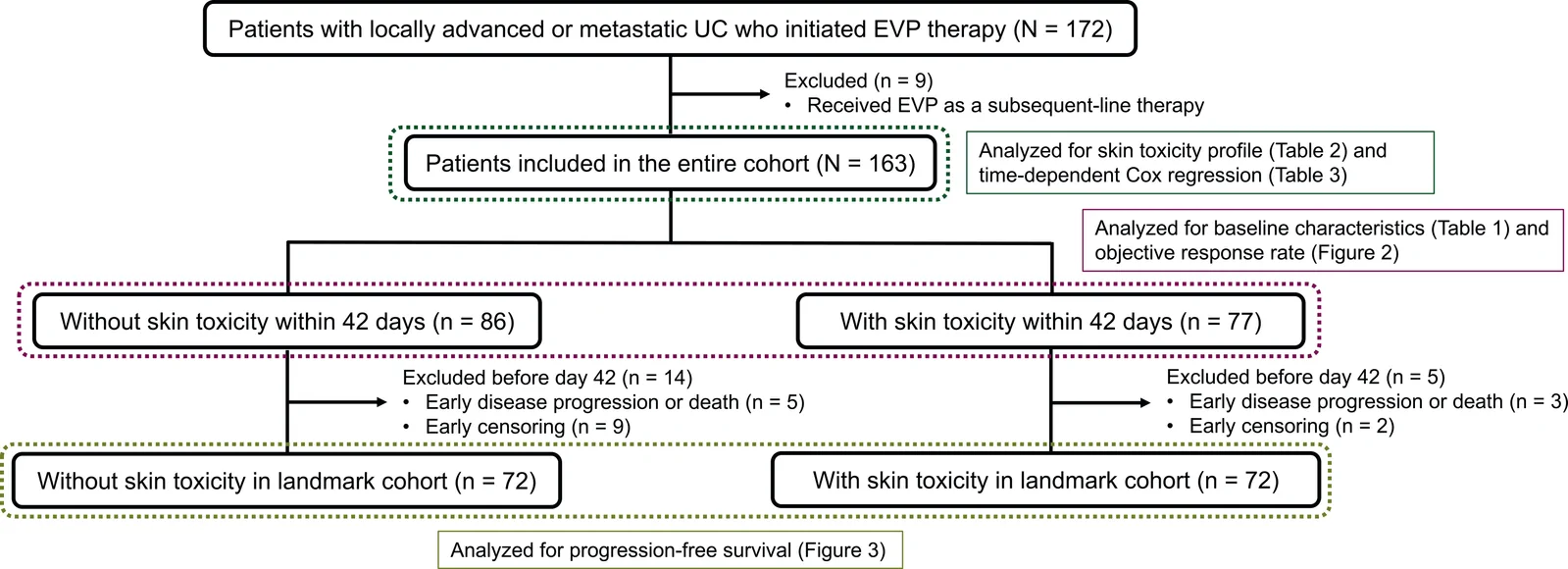
**Patient selection flowchart, illustrating the patient selection process and stratification into specific analytical cohorts.** Following the exclusion of patients who received EVP as a subsequent-line therapy, the entire cohort (*N* = 163) was established to evaluate the skin toxicity profile and time-dependent Cox regression. This cohort was then stratified according to the development of skin toxicity within 42 days for the analyses of baseline characteristics and objective response rate. For progression-free survival analysis, a 42-day landmark cohort (*N* = 144) was defined by excluding patients who experienced early disease progression, died, or were censored before day 42. Colored dashed boxes indicate the specific subcohorts used for each corresponding analysis. EVP, enfortumab vedotin plus pembrolizumab; UC, urothelial carcinoma.

Baseline characteristics stratified by skin toxicity status within 42 days are summarized in Table 1. Patient characteristics were generally balanced between groups, except for the initial EV dose. The proportion receiving the standard initial EV dose was significantly higher among patients with skin toxicity than among those without (81.8% versus 65.1%, *P* = 0.021). Sex, age, ECOG PS, renal function, primary tumor site, histology, prior treatment, and metastatic sites were comparable between groups.

**Table 1:** Baseline patient characteristics stratified by the development of skin toxicity within 42 days

Characteristic	Without skin toxicity (*n* = 86)	With skin toxicity (*n* = 77)	*P* value^a^
Sex			
Male	61 (70.9)	60 (77.9)	0.37
Female	25 (29.1)	17 (22.1)	
Age at start of EVP therapy (years)	75.5 (70.0-80.0)	73.0 (69.0-78.0)	0.08
BMI (kg/m^2^)	22.2 (19.4-24.8)	22.8 (20.1-25.3)	0.45
ECOG performance status			
0-1	76 (88.4)	72 (93.5)	0.29
≥2	10 (11.6)	5 (6.5)	
Diabetes mellitus	20 (23.3)	18 (23.4)	1.00
eGFR <30 ml/min/1.73 m^2^	12 (14.0)	9 (11.7)	0.82
Primary site			
Bladder	51 (59.3)	45 (58.4)	0.31
Pelvis or ureter	32 (37.2)	25 (32.5)	
Both	3 (3.5)	7 (9.1)	
Histology^b^			
Pure UC	64 (77.1)	66 (86.8)	0.15
Variant histology involved	19 (22.9)	10 (13.2)	
Not evaluated	3	1	
Treatment history before EVP			
None (e.g. *de novo* locally advanced or metastatic)	51 (59.3)	46 (59.7)	0.32
Primary tumor resection	25 (29.1)	27 (35.1)	
Primary preservation therapy	10 (11.6)	4 (5.2)	
Prior adjuvant nivolumab therapy	8 (9.3)	4 (5.2)	0.38
Metastatic sites			
Lymph node	55 (64.0)	51 (66.2)	0.87
Lung	33 (38.4)	21 (27.3)	0.14
Bone	13 (15.1)	13 (16.9)	0.83
Liver	11 (12.8)	6 (7.8)	0.32
Initial EV dose			
Standard dose (1.25 mg/kg)	56 (65.1)	63 (81.8)	0.021
Reduced dose (<1.25 mg/kg)	30 (34.9)	14 (18.2)	

Values are given as *n* (%) or median (IQR).

BMI, body mass index; ECOG, Eastern Cooperative Oncology Group; eGFR, estimated glomerular filtration rate; EV, enfortumab vedotin; EVP, enfortumab vedotin plus pembrolizumab; IQR, interquartile range; UC, urothelial carcinoma.

a *P* values were calculated using Fisher’s exact test for categorical variables and the Mann–Whitney *U* test for continuous variables.

b Patients with not evaluated histology were excluded from the statistical comparison for this variable.

### Clinical profile of skin toxicity

Skin toxicity occurred in 94 of 163 patients (57.7%) during the overall treatment course, with grade ≥3 events observed in 15 (9.2%). Among the affected patients, most events were grade 1-2 (84.0%), whereas grade ≥3 events accounted for 16.0%. The median time to onset was 13.0 days (IQR, 8.0-22.0 days) (Table 2).

**Table 2:** Clinical profile and management of EVP-related skin toxicity

Clinical characteristics	Total (*n* = 94)
Highest severity grade	
Grade 1-2	79 (84.0)
Grade ≥3	15 (16.0)
Time to onset (days)	13.0 (8.0-22.0)
Management^a^	
Topical corticosteroids	77 (81.9)
Emollients	11 (11.7)
Systemic antihistamines	41 (43.6)
Systemic corticosteroids	9 (9.6)
Action taken due to skin toxicity	
EV dose reduction	22 (23.4)
EV interruption	16 (17.0)
EV discontinuation	2 (2.1)
Pembrolizumab discontinuation	1 (1.1)
Recovery status	
Recovered	81 (86.2)
Not recovered	9 (9.6)
Not evaluated	4 (4.3)
Time to resolution (days)^b^	22.0 (9.5-34.5)

Values are given as *n* (%) or median (IQR).

EV, enfortumab vedotin; EVP, enfortumab vedotin plus pembrolizumab; IQR, interquartile range.

a Patients can receive more than one type of management for skin toxicity.

b Calculated for patients who achieved recovery.

Topical corticosteroids were the most frequently used management strategy (81.9%), followed by systemic antihistamines (43.6%), emollients (11.7%), and systemic corticosteroids (9.6%). Management strategies for skin toxicity included EV dose reduction in 22 patients (23.4%) and EV interruption in 16 (17.0%). Permanent discontinuation was required in two patients (2.1%) for EV and one (1.1%) for pembrolizumab. Recovery was observed in 81 patients (86.2%), with a median recovery time of 22.0 days (IQR, 9.5-34.5 days).

### ORRs according to skin toxicity

The best overall response according to the development of skin toxicity within 42 days is shown in Figure 2. The ORR was significantly higher in patients with skin toxicity than in those without skin toxicity (70.1% versus 44.2%, *P* < 0.001). CR was achieved in 12 patients (15.6%) with skin toxicity and 6 (7.0%) without skin toxicity. PR was observed in 42 (54.5%) and 32 (37.2%) patients, respectively. Progressive disease was less frequent in patients with skin toxicity than in those without (13.0% versus 20.9%). In a sensitivity analysis restricted to patients assessable at the 42-day landmark (*n* = 144), the ORR remained significantly higher in patients with skin toxicity than in those without skin toxicity (75.0% versus 52.8%, *P* = 0.009).

**Figure 2:**
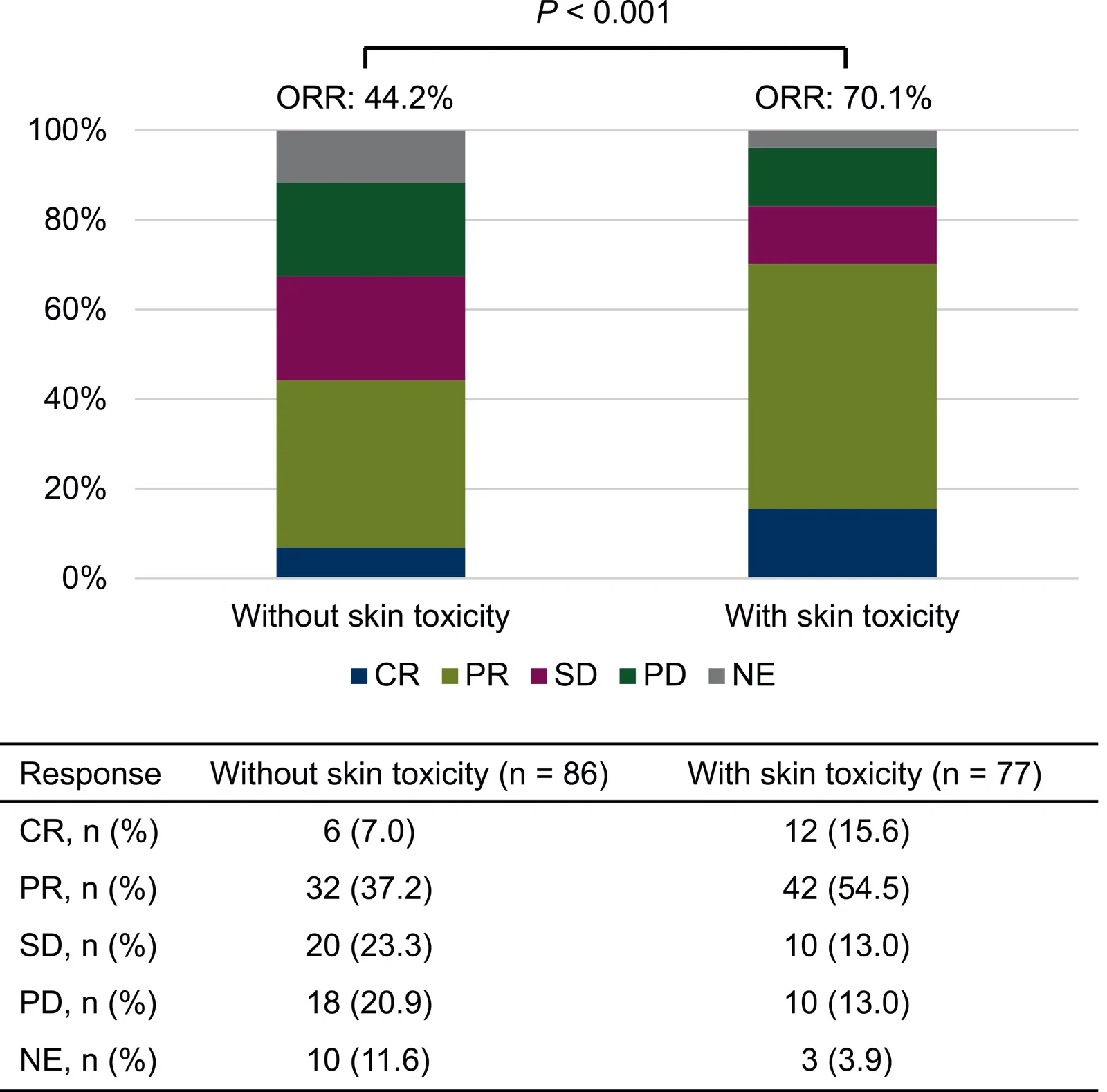
**Best overall response according to the development of skin toxicity within 42 days (*N* = 163).** The stacked bar chart and corresponding table show the frequency and percentage of patients in each response category stratified by the presence (*n* = 77) or absence (*n* = 86) of skin toxicity that developed within 42 days of treatment initiation. The ORR was defined as the proportion of patients who achieved a CR or PR. *P* values were calculated using Fisher’s exact test. CR, complete response; NE, not evaluable; ORR, objective response rate; PD, progressive disease; PR, partial response; SD, stable disease.

### Association between skin toxicity and survival outcomes

Among the 163 patients, 47 PFS events were observed. In the 42-day PFS landmark cohort (*n* = 144), 39 PFS events occurred, including 25 among 72 patients without skin toxicity and 14 among 72 patients with skin toxicity. Kaplan–Meier analysis demonstrated that patients who developed skin toxicity had significantly longer PFS than those without skin toxicity (HR 0.43, 95% CI 0.22-0.84, log-rank *P* = 0.011) (Figure 3). In an exploratory analysis according to skin toxicity severity, PFS differed significantly among patients with no skin toxicity, grade 1-2 toxicity, and grade ≥3 toxicity (log-rank *P* = 0.010), although no clear severity-dependent gradient was observed (Supplementary Figure S1, available at https://doi.org/10.1016/j.esmorw.2026.100804).

**Figure 3:**
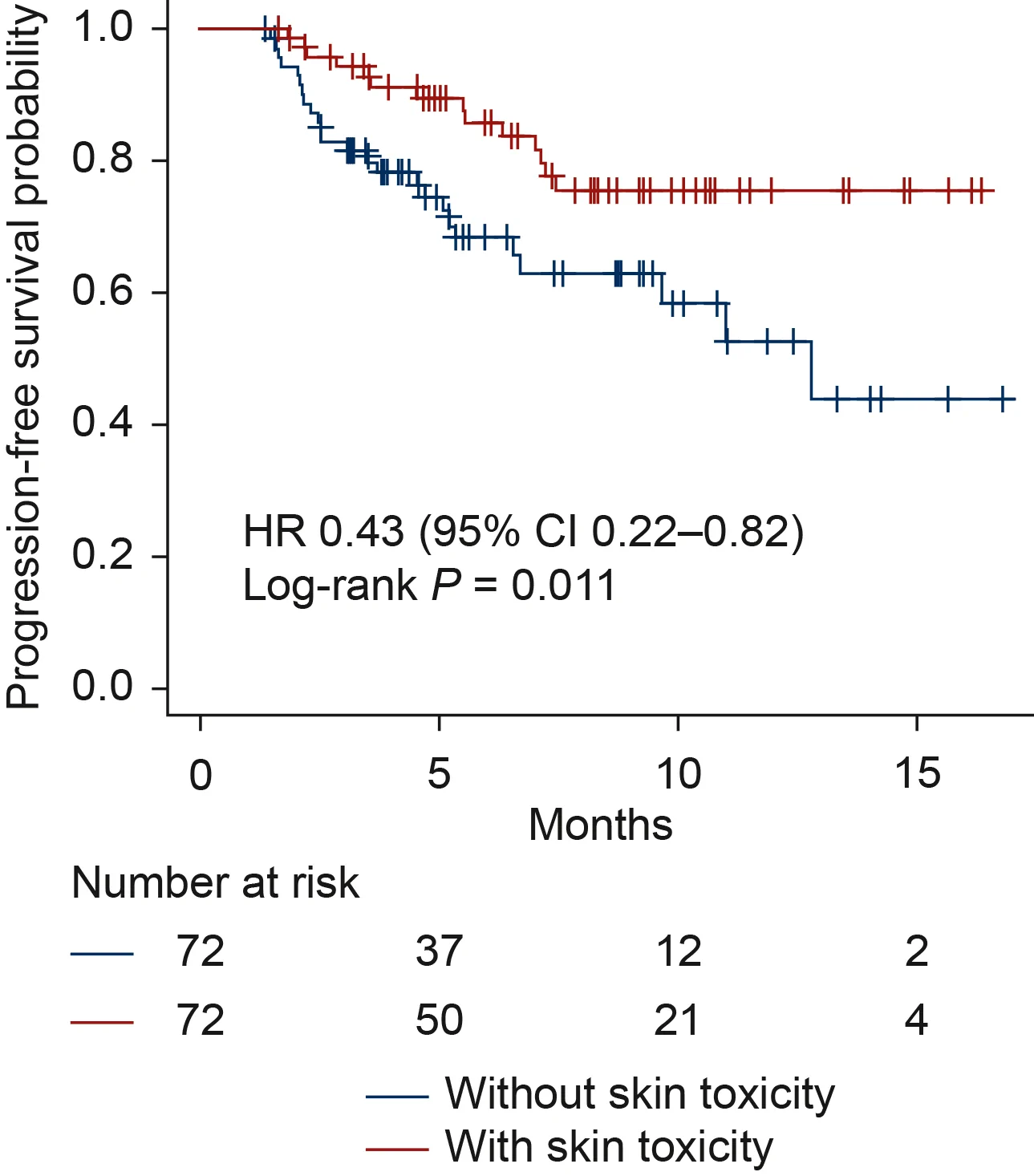
**Kaplan–Meier estimates of PFS according to the development of skin toxicity in a 42-day landmark analysis.** Kaplan–Meier curves comparing PFS between patients with and without skin toxicity in the 42-day landmark cohort. Patients who experienced disease progression, died, or were censored before day 42 were excluded (*N* = 144). CI, confidence interval; HR, hazard ratio; PFS, progression-free survival.

As a complementary approach to account for immortal time bias, a time-dependent Cox regression analysis was carried out (Table 3). In univariable analysis, the development of skin toxicity was significantly associated with improved PFS (HR 0.36, 95% CI 0.19-0.66, *P* < 0.001). In the multivariable model adjusted for prespecified clinically relevant covariates, the development of skin toxicity remained independently associated with favorable PFS (HR 0.44, 95% CI 0.23-0.83, *P* = 0.011). Prior adjuvant nivolumab therapy was independently associated with worse PFS (HR 3.61, 95% CI 1.49-8.78, *P* = 0.005). In a sensitivity analysis additionally adjusting for the initial EV dose, skin toxicity remained significantly associated with longer PFS (HR 0.38, 95% CI 0.20-0.73, *P* = 0.004), while reduced initial EV dosing was also associated with longer PFS (HR 0.46, 95% CI 0.21-0.99, *P* = 0.048) (Supplementary Table S1, available at https://doi.org/10.1016/j.esmorw.2026.100804).

**Table 3:** Univariable and multivariable time-dependent Cox regression analyses for PFS (*N* = 163)

Parameter	Univariable analysis		Multivariable analysis	
	HR (95% CI)	*P* value	HR (95% CI)	*P* value
Sex (female versus male)	1.52 (0.82-2.82)	0.18		
Age (≥75 versus <75 years)	1.66 (0.92-3.01)	0.094		
ECOG PS (≥2 versus 0-1)	2.27 (0.96-5.36)	0.062	1.79 (0.71-4.50)	0.22
eGFR (<30 versus ≥30)	0.89 (0.35-2.26)	0.81		
Primary site (upper tract involved versus bladder)	0.97 (0.54-1.73)	0.91		
Histology (variant histology versus pure UC)	1.79 (0.88-3.65)	0.11	1.67 (0.79-3.56)	0.18
Prior treatment (received versus none)	1.05 (0.59-1.87)	0.87		
Prior adjuvant nivolumab (yes versus no)	2.76 (1.17-6.50)	0.021	3.61 (1.49-8.78)	0.005
Liver metastasis (yes versus no)	2.34 (1.09-5.05)	0.030	2.02 (0.86-4.75)	0.11
Initial EV dose (reduced versus standard)	0.66 (0.33-1.31)	0.23		
Skin toxicity (yes versus no)	0.36 (0.19-0.66)	<0.001	0.44 (0.23-0.83)	0.011

CI, confidence interval; ECOG PS, Eastern Cooperative Oncology Group performance status; eGFR, estimated glomerular filtration rate; EV, enfortumab vedotin; HR, hazard ratio; PFS, progression-free survival; UC, urothelial carcinoma.

In the exploratory OS analysis, Kaplan–Meier analysis of the 42-day landmark cohort (*n* = 148) demonstrated significantly longer OS in patients who developed skin toxicity than in those without skin toxicity (HR 0.35, 95% CI 0.13-0.94; log-rank *P* = 0.029) (Supplementary Figure S2, available at https://doi.org/10.1016/j.esmorw.2026.100804). Consistently, univariable time-dependent Cox regression showed that skin toxicity was associated with a lower risk of death (HR 0.32, 95% CI 0.13-0.76. *P* = 0.010).

## Discussion

In this prospective, multicenter observational study, we evaluated the clinical significance of skin toxicity in patients with la/mUC receiving first-line EVP therapy in a real-world setting. The most notable finding was that skin toxicity was associated with favorable treatment outcomes. Patients who developed skin toxicity had a higher ORR; although this association may have been influenced by the shorter opportunity to develop skin toxicity among patients with early disease progression, it persisted in a sensitivity analysis restricted to patients assessable at the 42-day landmark. Skin toxicity was also associated with longer PFS in the landmark analysis and this association was further supported by the time-dependent Cox model. Exploratory analyses also suggested an association between skin toxicity and longer OS. These findings suggest that skin toxicity may serve as a potential on-treatment indicator of favorable outcomes.

Skin toxicity is a common adverse event associated with EVP. In EV-302, any-grade and grade ≥3 skin reactions were reported in 66.8% and 15.5% of patients, respectively, whereas real-world cohorts have reported any-grade skin toxicity in 57.0%-60.4% of patients.^1^^,^^4^^,^^5^ In this study, the incidences of any-grade skin toxicity (57.7%) and grade ≥3 events (9.2%) were broadly consistent with previous findings. Importantly, EV dose reduction and treatment interruption due to skin toxicity were required in 23.4% and 17.0% of affected patients, respectively, whereas permanent discontinuation of EV or pembrolizumab was rare. This is consistent with recent real-world data on EV ± pembrolizumab showing that skin toxicity often required treatment interruption or dose reduction (each 46%) but rarely led to treatment discontinuation (5%).^15^ In our cohort, corticosteroids were used therapeutically after the development of skin toxicity, primarily as topical treatment (81.9%), whereas systemic corticosteroids were required in only 9.6% of affected patients. Together with the 86.2% recovery rate in our cohort, these findings support the management of EVP-associated skin toxicity with early supportive care and appropriate dose modification.

As EVP consists of both EV and pembrolizumab, skin toxicity should be interpreted as a composite clinical phenomenon. Although immune checkpoint inhibitor-related skin events can occur early, programmed cell death protein-1 inhibitor-related cutaneous reactions tend to develop later (median onset 4.2 months).^16^ By contrast, EV-related skin toxicity is typically observed early, mainly within the first two cycles (median onset, 0.4-0.6 months).^6^^,^^17^^,^^18^ The median onset of 13.0 days in our cohort is therefore consistent with a substantial contribution from EV-related toxicity during the early phase of EVP therapy. However, individual clinical phenotypes were not systematically recorded and EV-related skin toxicity could not be reliably distinguished from pembrolizumab-related skin events.

Studies of EV monotherapy have consistently reported associations between skin toxicity and favorable clinical outcomes.^6^, ^7^, ^8^ These findings suggest that skin toxicity may provide clinically observable on-treatment prognostic information, although its role as a predictive biomarker has not been established. A similar relationship between treatment-related toxicity and clinical efficacy has been observed with vascular endothelial growth factor-targeted tyrosine kinase inhibitors in renal cell carcinoma, in which treatment-induced hypertension has been associated with favorable outcomes.^19^ In the case of EV, one possible biological explanation involves nectin-4, a therapeutic target of EV that is widely expressed in UC and normal skin tissues.^9^ Previous studies have shown that nectin-4 expression contributes to EV sensitivity, whereas heterogeneous or reduced membranous nectin-4 expression is associated with decreased efficacy.^9^^,^^20^^,^^21^ Recent imaging studies have also demonstrated heterogeneous nectin-4 expression across metastatic sites in UC.^22^ These observations raise the hypothesis that skin toxicity may partly reflect EV exposure and on-target activity in nectin-4-expressing tissues. However, EV pharmacokinetics, cumulative exposure, and nectin-4 expression were not evaluated in our study. Although the early median onset of skin toxicity (13 days) is consistent with a contribution from EV-related toxicity, it does not establish this mechanism. Moreover, the exploratory analysis by toxicity severity showed no clear toxicity–efficacy gradient, with the most favorable PFS observed in patients with grade 1-2 toxicity. Therefore, this proposed relationship remains hypothetical.

Interestingly, patients receiving the standard initial EV dose developed skin toxicity more frequently. Previous studies of EV monotherapy have reported an association between higher EV dose and the development of skin toxicity,^23^^,^^24^ whereas another study did not demonstrate an association between EV dose and either PFS or OS.^6^ These findings suggest that EV dose may influence skin toxicity, whereas its relationship with antitumor efficacy is less clear. In our study, the initial EV dose was determined at the treating physician’s discretion and could, therefore, have reflected patient frailty, comorbidities, physician judgment, or other unmeasured factors. Importantly, in a sensitivity analysis additionally adjusting for the initial EV dose, the association between skin toxicity and longer PFS remained consistent. Although reduced initial dosing was also associated with longer PFS in this analysis, this finding should be interpreted cautiously because an association between initial dose and PFS was not demonstrated in univariable analysis and may reflect patient selection or residual confounding.

Another notable observation was that prior adjuvant nivolumab therapy was independently associated with shorter PFS. However, this finding should be interpreted with caution because only 12 patients had previously received adjuvant nivolumab. Nevertheless, this issue is clinically relevant because relapses after adjuvant immunotherapy are expected to become more common as such therapy becomes the standard treatment for high-risk muscle-invasive UC. Although the underlying mechanism remains unclear, prior nivolumab exposure may reflect more aggressive disease biology or reduced sensitivity to the pembrolizumab component of EVP. Further studies are needed to clarify the effect of prior adjuvant nivolumab on EVP efficacy and to optimize treatment strategies.

Our findings have several clinical implications. As treatment-emergent toxicities are inherently time dependent, analyses based solely on toxicity status are susceptible to immortal time bias. The concordant findings from the prespecified 42-day landmark and time-dependent Cox analyses, therefore, support the robustness of the observed association. Clinically, skin toxicity may provide on-treatment prognostic information regarding favorable outcomes; however, this association should not be interpreted as evidence against clinically indicated dose modification. Favorable outcomes were observed despite EV dose reduction or treatment interruption when required for toxicity management. Proactive monitoring and timely dermatological management remain important to prevent progression to severe events, guide appropriate dose modification according to toxicity severity and established recommendations, and maintain treatment continuity.

This study had several limitations. Firstly, although this was a prospective multicenter study, it was not randomized and residual confounding cannot be excluded. In particular, the initial EV dose was determined at the treating physician’s discretion and may have reflected patient frailty, clinical judgment, or other unmeasured factors. In addition, only 12 patients had prior adjuvant nivolumab exposure; therefore, its association with shorter PFS should be also interpreted with caution. Secondly, the median follow-up duration was relatively short (7.1 months) and the OS data were immature; therefore, the exploratory OS findings should be interpreted cautiously and the impact of skin toxicity on long-term survival remains to be determined. Thirdly, tumor responses were assessed without an independent central radiological review. Although skin toxicity was prospectively assessed, mild events, particularly grade 1 toxicity, may have been underreported, and differences in recognition, documentation, and grading across participating institutions and treating physicians cannot be excluded. Finally, distinguishing between EV-related skin toxicity and pembrolizumab-related immune-mediated skin reactions in routine clinical practice is challenging. Moreover, nectin-4 expression and pharmacokinetic parameters were not evaluated, limiting the mechanistic interpretation.

### Conclusions

This prospective multicenter observational study demonstrated that skin toxicity associated with first-line EVP was frequent, occurred early, and was generally manageable. Its development was associated with a higher ORR and longer PFS. The association with PFS persisted after accounting for immortal time bias. Skin toxicity may serve as a potential on-treatment prognostic indicator of favorable outcomes in patients receiving EVP. Longer follow-up and external validation are needed to confirm its clinical significance.

## Declaration of AI and AI-assisted technologies in the writing process

During the preparation of this work, the authors used ChatGPT (OpenAI) and Gemini (Google) in order to improve the readability and language of the manuscript. After using these tools, the authors reviewed and edited the content as needed and take full responsibility for the content of the publication.

## Supplementary data

Supplementary Table S1

Supplementary_Figures_ESMORW_100804

Supplementary_table_1_ESMORW_100804
