# Supplementary material for: Skin toxicity and effectiveness of first-line enfortumab vedotin plus pembrolizumab for urothelial carcinoma: a prospective multicenter real-world study in Japan
Source: ESMO Real World Data Digit Oncol. 2026 Oct 17;14:100804. doi: 10.1016/j.esmorw.2026.100804 (PMC13695236; doi:10.1016/j.esmorw.2026.100804)
Supplement: Supplementary file 1 [file mmc1.pdf]

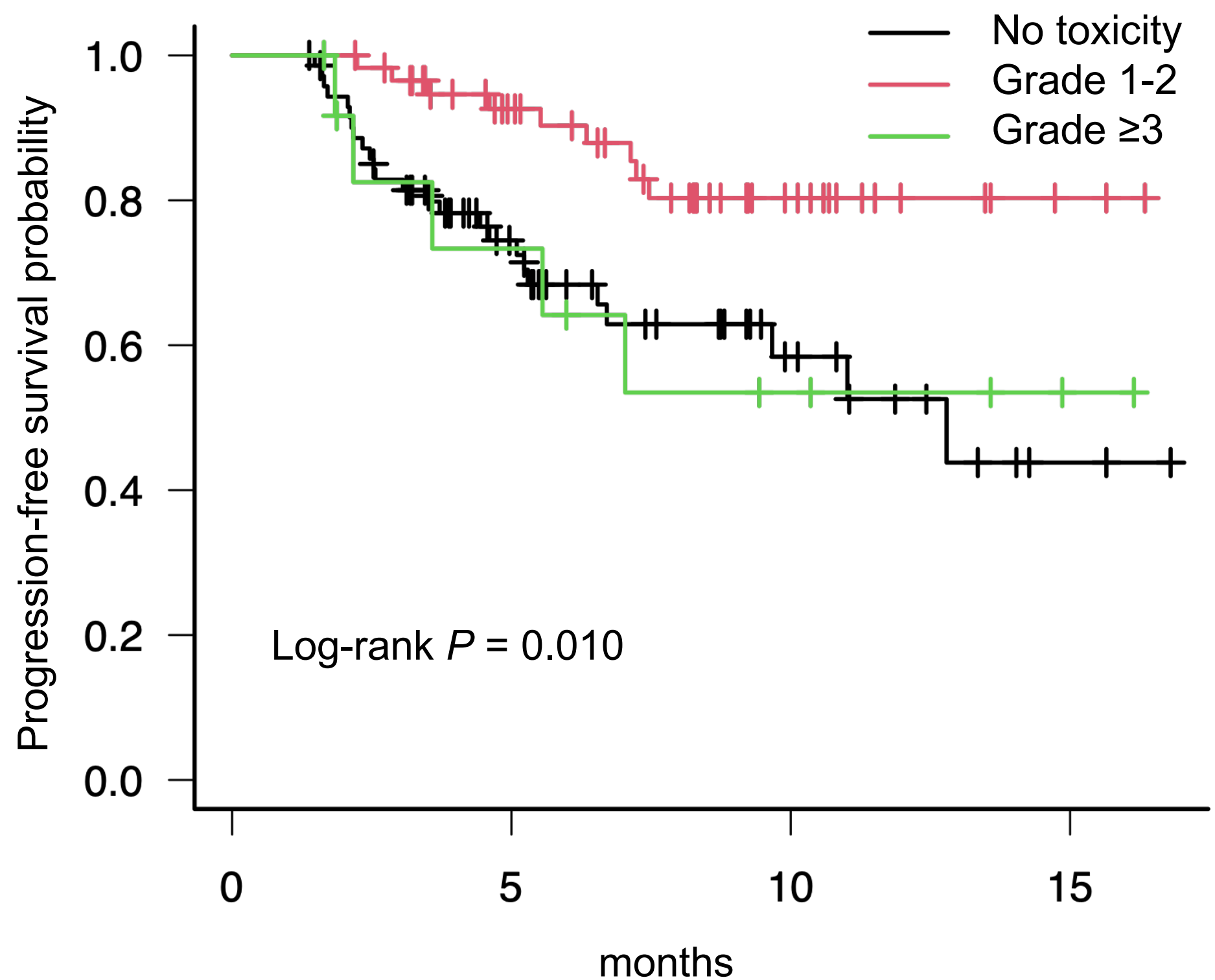

Number at risk

No toxicity	72	37	12	2
Grade 1-2	59	42	17	3
Grade ≥3	13	8	4	1
