# Supplementary material for: Skin toxicity and effectiveness of first-line enfortumab vedotin plus pembrolizumab for urothelial carcinoma: a prospective multicenter real-world study in Japan
Source: ESMO Real World Data Digit Oncol. 2026 Oct 17;14:100804. doi: 10.1016/j.esmorw.2026.100804 (PMC13695236; doi:10.1016/j.esmorw.2026.100804)
Supplement: Supplementary file 3 [file mmc3.docx]

**Supplementary Figure 1. Kaplan–Meier estimates of progression-free survival according to skin toxicity severity in the 42-day landmark cohort.**

Patients were classified as having no skin toxicity (n = 72), grade 1–2 toxicity (n = 59), or grade ≥3 toxicity (n = 13) according to the maximum grade observed within 42 days.

**Supplementary Figure 2. Kaplan-Meier estimates of overall survival according to the development of skin toxicity in a 42-day landmark analysis.**

Kaplan–Meier curves comparing overall survival between patients with and without skin toxicity in the 42-day landmark cohort. Patients who died or were censored before day 42 were excluded (n = 148). Abbreviations: HR, hazard ratio.
