# Supplementary material for: Skin toxicity and effectiveness of first-line enfortumab vedotin plus pembrolizumab for urothelial carcinoma: a prospective multicenter real-world study in Japan
Source: ESMO Real World Data Digit Oncol. 2026 Oct 17;14:100804. doi: 10.1016/j.esmorw.2026.100804 (PMC13695236; doi:10.1016/j.esmorw.2026.100804)
Supplement: Supplementary file 4 [file mmc4.docx]

**Supplementary Table 1. Sensitivity analysis using a multivariable time-dependent Cox regression model for progression-free survival including initial EV dose (N = 163).**

|  | Multivariable analysis | |
| --- | --- | --- |
|  | HR (95% CI) | *P* value |
| ECOG PS (≥2 vs. 0-1) | 1.93 (0.78–4.80) | 0.16 |
| Histology (variant histology vs. pure UC) | 1.76 (0.83–3.73) | 0.14 |
| Prior adjuvant nivolumab (yes vs. no) | 2.98 (1.21–7.34) | 0.018 |
| Liver metastasis (yes vs. no) | 1.93 (0.82–4.53) | 0.13 |
| Initial EV dose (reduced vs. standard) | 0.46 (0.21–0.99) | 0.048 |
| Skin toxicity (yes vs. no) | 0.38 (0.20–0.73) | 0.004 |

Abbreviations: CI, confidence interval; ECOG PS, Eastern Cooperative Oncology Group performance status; EV, enfortumab vedotin; HR, hazard ratio; UC, urothelial carcinoma.
